# The diabetes mellitus multimorbidity network in hospitalized patients over 50 years of age in China: data mining of medical records

**DOI:** 10.1186/s12889-024-18887-y

**Published:** 2024-05-29

**Authors:** Chao Chen, Xueting Zheng, Shaobing Liao, Shimin Chen, Minyi Liang, Kang Tang, Mingjuan Yin, Huansheng Liu, Jindong Ni

**Affiliations:** 1 Precision Key Laboratory of Public Health, School of Public Health, Guangdong Medical University, No.1 Xincheng Road, Songshan Lake, Dongguan, 523808 Guangdong China; 2 Department of Epidemiology and Health Statistics, School of Public Health, Guangdong Medical University, Dongguan, Guangdong China; 3 Maternal and Child Research Institute, Shunde Women and Children’s Hospital, Guangdong Medical University, Foshan, China

**Keywords:** Diabetes mellitus, Multimorbidity, Aging, Association rule mining, Non-communicable diseases

## Abstract

**Objective:**

Many diabetes mellitus (DM) patients suffer from multimorbidity. Understanding the DM multimorbidity network should be given priority. The purpose of this study is characterize the DM multimorbidity network in people over 50 years.

**Methods:**

Data on 75 non-communicable diseases (NCDs) were extracted from electronic medical records of 309,843 hospitalized patients older than 50 years who had at least one NCD. The association rules analysis was used as a novel classification method and combined with the Chi-square tests to identify associations between NCDs and DM.

**Result:**

A total of 12 NCDs were closely related to DM, {cholelithiasis, DM} was an unexpected combination. {dyslipidemia, DM} and {gout, DM} had the largest $\text{lift}$ in the male and female groups, respectively. The negative related group included 7 NCDs. There were 9 NCDs included in the strong association rules. Most combinations were different by age and sex. In males, the strongest rule was {peripheral vascular disease (PVD), dyslipidemia, DM}, while {hypertension, dyslipidemia, chronic liver disease (CLD), DM} was the strongest in females. In patients younger than 70 years, hypertension, CLD, and dyslipidemia were the most dominant NCDs in the DM multimorbidity network. In patients 70 years or older, chronic kidney disease (CKD), CVD, CHD, and heart disease (HD) frequently co-occurred with DM.

**Conclusion:**

Future primary healthcare policies for DM should be formulated based on age and sex. In patients younger than 70 years, more attention to hypertension, CLD, and dyslipidemia is required, while attention to CKD, CVD, CHD and HD is needed in patients older than 70 years.

**Supplementary Information:**

The online version contains supplementary material available at 10.1186/s12889-024-18887-y.

## Introduction

### Background

The global prevalence of DM in adults is on the rise: in 2017 it was 8.8% and it is expected to rise to 9.9% by 2045. In addition to diagnosed DM, approximately 352 million people worldwide are at risk of developing DM or pre-DM, and that number is expected to rise to 532 million by 2045 [1]. China has one of the largest DM populations in the world. In 2017, there were 425 million adults with DM worldwide, of which 114 million (more than a quarter) were from China. The number of adults with DM in China is expected to rise to 120 million in 2045 [2]. Moreover, many DM patients suffer from at least one additional disease called multimorbidity. It means two or more NCDs co-occur in a patient [3]. Multimorbidity affects more than half of the elderly population and almost all hospitalized geriatric patients [4]. The coexistence of NCDs in DM patients is more than a random event. Typically, it is due to the causal relationship between some NCDs or a shared pathogenic factor [5, 6]. Therefore, prevention and treatment of DM with multimorbidity are very important. Some studies aimed to identify the multimorbidity patterns in patients and confirmed the existence of clinically plausible multimorbidity patterns that evolve over time [7, 8]. Unfortunately, recommended management approaches for multimorbidity in patients with DM are lacking in most practice guidelines [9].

There is a difference in the etiological analysis of patients with a single NCD and those with multimorbidity [10]. Most clinical practice guidelines and healthcare training and delivery focus on a single NCD, leading to care that is sometimes inadequate[11] and often results in an increase of intervention measures, such as numerous hospital visits, and polypharmacy [12, 13]. Consequently, the current healthcare systems fail to appropriately address the healthcare needs of patients with multimorbidity.

Although multimorbidity has been introduced in policy and practice in developed countries, developing countries have not considered it a matter of public health urgency [14]. Improving the health status and quality of life of people affected by multimorbidity requires a new integrated and innovative treatment model [15]. We aimed to explore the interrelationships in the DM multimorbidity network. It may help to address the challenge and provide new insights for interventions in DM multimorbidity.

Occurrences of multimorbidity in DM patients has been recognized and investigated in previous studies [12, 16–20]. Cluster analysis [12, 16, 19], network analysis [15, 17, 18, 20, 21], and latent class analysis [22, 23] were used to explore the multimorbidity patterns. However, many of these studies were limited either by their small sample sizes [15, 17, 19, 24, 25] or by the small number of NCDs [17, 19, 22, 24, 26] used to study multimorbidity patterns. These previous studies provided limited information on the DM multimorbidity network.

### Objectives

We presented the DM multimorbidity network in middle-aged and older adults and used association rules mining (ARM) to explore the relationship between 74 NCDs and DM. We focused on examining patterns that are present in people with DM. The first step was to investigate whether there were any associations between 74 NCDs and DM. The $lift$ generated by the ARM was used as a classification indicator to identify the relationship between 74 NCDs and DM. The Chi-square test was used to test the statistical significance of the associations between the NCDs and DM. The second step was to explore the DM multimorbidity network and assess the variations in these patterns by age and sex. Based on the ARM algorithm, which can fully consider the importance and correlation strength between the NCDs and DM, we can obtain the multimorbidity patterns of DM.

## Methods

### Data source

The original data was obtained from the homepages of inpatient medical records through the Shenzhen National Health Information Platform, a data center that collects medical cases information from all medical institutions in Shenzhen. All inpatient records from January 1, 2017 to December 31, 2018 were included. Data include demographic characteristics of hospitalized patients (sex and age), information on inpatient diagnoses of NCDs and information on personal identifiers. All diagnoses were coded according to International Classification of Disease version 10 (ICD-10). First, we extracted a total of more than 3 million records with age ≥ 49 years in 2017 and age ≥ 50 years in 2018. Second, according to ICD-10, patients were excluded if they had not been diagnosed with at least one of the NCDs. Patients were also excluded if they had incomplete information, such as sex, age and personal identification. After matching information on personal identifiers, if the same personal identifiers appeared in both 2017 and 2018, the age in 2018 was used. The 49-year-old patients who only appeared in 2017 were deleted. Then the same personal identifiers were merged. The NCDs were selected based on those most frequently mentioned in previous studies of multimorbidity[16, 18, 19, 21]. On the other hand, NCDs with a proportion larger than 0.001 were selected, as they were considered to have a significant impact on the long-term management and quality of life of middle-aged and older Chinese individuals. Finally, we included data from a total of 309,843 participants aged ≥ 50 years with at least one of the 75 NCDs. Supplementary Material 1 lists all NCDs included and their corresponding ICD-10 codes.

### Defining multimorbidity

Multimorbidity was defined as concurrently suffering from two or more NCDs [13]. NCDs were identified if they had been documented using inpatient ICD-10 codes in individual recent medical records from January 1, 2017 to December 31, 2018. To explore the DM multimorbidity network more comprehensively, we included 75 NCDs to study the DM multimorbidity network based on previous studies and the current data of this study.

### Statistical analyses

#### Descriptive analysis

Patients were categorized into four subgroups according to age (50 − 59, 60 − 69, 70 − 79, and ≥ 80 years). Sex was categorized into two subgroups and cross-combined with age into eight age-sex subgroups. Descriptive statistics, including number and proportion (%), were used in the study population. The Chi-square test was performed to compare differences in the characteristics of patients with and without DM. Age was presented as median (interquartile range; IQR). The 10 most frequent dyads, triads and quartets of NCDs combined with DM by sex and age were evaluated. A *P* < 0.05 was considered statistically significant. All the descriptive statistical analyses in this study were performed using R 3.4.0 (The R Foundation for Statistics and Mathematics, Vienna, Austria). ARM was performed using R 3.4.0 with the arules package.

#### Association rule mining

ARM is used to examine associations between NCDs [17, 27, 28]. It is a fast method to discover combinations of NCDs that occur more frequently than expected and might provide insights into NCDs and aging mechanisms. Several applications of ARM in the medical domain include examining disease co-occurrences [12, 15, 16], identifying adverse effects of drugs [29], and detecting risk factors for disease [30–34]. We analyzed the data using the Apriori algorithm and applied the ARM to determine the common multimorbidity patterns for DM that met a minimum requirement of measurement indicators.

The three commonly used measurement ratios were used. The support ($sup$) is a measure of how frequently NCD $A$ and NCD $B$ combinations appear in the dataset. It measures the importance of rule $\left\{A,B\right\}$ and is defined as: $sup\left(AB\right)=P(AB)$. A higher $sup$ indicates that the rule is more important, and it is usually needed to set a minimum threshold to exclude rules that are not important. The confidence ($con$) is the conditional probability that a participant who has NCD $A$ also has NCD $B$, and it is defined as: $con\left(AB\right)=P\left(B|A\right)=P(AB)/P(A)$. The $lift(AB)$ is the ratio of the observed $sup(AB)$ to that expected if $A$ and $B$ are independent. It is defined as: $lift\left(AB\right)=P(AB)/(P\left(A\right)P\left(B\right))=con\left(AB\right)/P(B)$ [28]. A higher $lift$ indicates a higher chance of co-occurrence of NCD $A$ with NCD $B$ and a more significant association. The $lift$ measures the strength of an association as a rule within ARM and is therefore considered the main outcome in this study. It can be used to identify rules whether the dependence between $A$ and $B$ is weak or strong [35]. We applied this method to examine the association in a dataset of people with DM and 74 other NCDs using a classifier based on the $lift$. When $lift\left(AB\right)=a>1$, this indicates that $A$ combined with $B$ occurs $a$-fold more than expected under statistical independence. It can be interpreted as a positive relationship between $A$ and $B$. When $lift\left(AB\right)<1$ indicates that the joint set $\left\{A,B\right\}$ appear less often than expected, there is a negative relationship between $A$ and $B$. When $lift\left(AB\right)=1$, this indicates that no association between $A$ and $B$. Hence, a higher $sup$ indicates a more important joint set $\left\{A,B\right\}$. A higher $lift$ indicates a stronger association of the joint set $\left\{A,B\right\}$. The $sup$, $con$, $lift$ are related to the effect size of associations, as opposed to simple tests of statistical significance [17]. Association rules with more than two items are similar to those with two items. The $sup({A}_{1}{A}_{2}\dots {A}_{n}B)$ ($n\ge 2$) indicates how frequently NCDs ${A}_{1}$, ${A}_{2}$,…, ${A}_{n}$ and NCD $B$ occur in the dataset. The $lift({A}_{1}{A}_{2}\dots {A}_{n}B)$ is the ratio of the observed $sup({A}_{1}{A}_{2}\dots {A}_{n}B)$ to that expected if $\{{A}_{1},{A}_{2},\dots ,{A}_{n}\}$ and $B$ are independent.

**Fig. 1:**
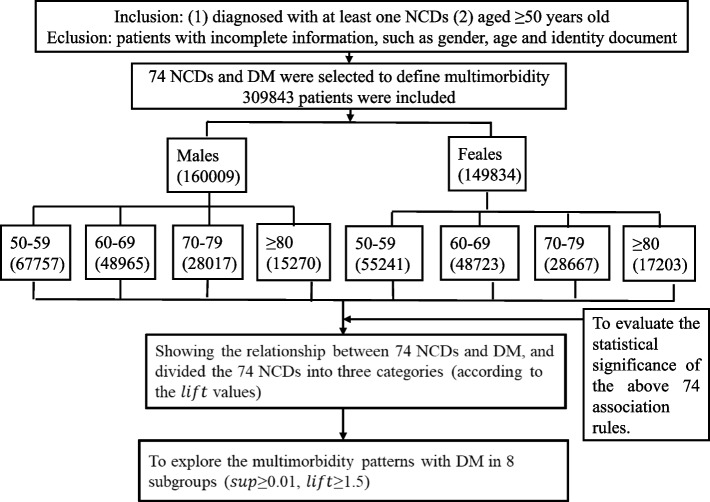
The flow chart of the main research steps

There were ${2}^{74}$ possible combinations for the 74 morbidities we included. Setting a higher threshold value would reduce the number of rules that might result in missing essential rules with low frequencies. Setting a lower threshold would prevent management from aggregating rules [36]. Appropriate $sup$ and $lift$ values help to mine reasonable rules and ensure the robustness of the model performance. Therefore, many rounds of testing and evaluation were carried out before defining final thresholds to mine reasonable rules and to ensure the robustness of the model performance. To avoid missing any critical association rule, we set the $sup$ threshold range from 0.001 to 0.01, increasing by 0.005 each time, and no minimum $con$ or $lift$ thresholds were limited. Since $lift(AB)=con(AB)/sup(A)$, the value of $con$ affects the value of $lift$. Other studies focus on the case of $lift>1$, so a high $con$ is accepted. However, we focus on $lift>1$, $lift=1$ and $lift<1$, so we do not limit the value of $con$. In our programme the minimum $con$ set as 0.0000001. In addition, using the objective indicator $lift$, we developed ARM as a novel classification method to examine associations between 74 NCDs and DM. It does not rely on preconceived assumptions about whether certain conditions are associated, thereby minimizing confirmation biases because no hypotheses were postulated [12] and is thus an objective parameter. The flow chart of the above analysis was shown in Fig. 1.

## Results

### Participants’ characteristics

The dataset consisted of 309,843 inpatients, of whom 149,834 (48.4%) were female and 160,009 (51.6%) were male. The median age was 63.0 years (IQR, 56.0 − 72.0) and that of men and women was 63 years (IQR, 55.0 − 71.0) and 64.0 years (IQR, 57.0 − 73.0), respectively. 21.1% (65,341/309,843) of participants had only one NCD, while 19.5% (60,399/309,843), 16.2% (50,286/309,843) and 13.3% (41,121/309,843) had two, three or four NCDs, respectively. The proportion of people with DM in the overall dataset was 22. 9% (70,932/309,843). 95.6% of hospitalized DM have two or more NCDs. Table 1 shows the proportion of people with DM in different age groups of men and women. The proportion of people with DM varied by sex and age (*P* < 0.001).

**Table 1:** Prevalence of DM in different age groups of males and females

Age	Female(*n*/%)	Male(*n*/%)	*P*-value
50–59	8739(15.8)	15,399(22.7)	< .001
60–69	11,558(23.7)	11,964(24.4)	< .001
70–79	8358(29.2)	7291(26.0)	< .001
≥ 80	4159(24.2)	3464(22.7)	< .001
Total	32,814(21.9)	38,118(23.8)	< .001

Tables 2 and 3 show the 10 most common dyads, triads and quartets of combinations of NCDs associated with DM by sex and age, respectively.

**Table 2:** Prevalence of the 10 most common morbidity about DM by sex

Type	Order	Total		Female		Male	
		NCDs	%	NCDs	%	NCDs	%
dyads	1	Hyp	14.2	Hyp	14.2	Hyp	14.2
	2	Dys	7.3	Dys	7.3	CD	7.4
	3	CD	7.0	CD	6.6	Dys	7.2
	4	CLD	6.2	CLD	5.6	CKD	6.8
	5	PVD	5.9	PVD	5.4	CLD	6.6
	6	CHD	5.7	CHD	5.1	PVD	6.3
	7	CKD	5.7	CKD	4.6	CHD	6.3
	8	HD	3.8	HD	3.3	PD	4.5
	9	gout	3.4	gout	3.0	HD	4.3
	10	Arr	2.7	Ane	2.5	gout	3.7
triads	1	Hyp,CD	5.6	Hyp,CD	5.4	Hyp,CD	5.7
	2	Hyp,Dys	4.8	Hyp,Dys	5.0	Hyp,CKD	4.7
	3	Hyp,CHD	4.4	Hyp,CHD	4.2	Hyp,CHD	4.7
	4	Hyp,PVD	4.3	Hyp,PVD	4.1	Hyp,Dys	4.6
	5	Hyp,CKD	4.1	Hyp,CLD	3.7	Hyp,PVD	4.5
	6	Hyp,CLD	3.9	Hyp,CKD	3.4	Hyp,CLD	4.0
	7	HD,CHD	3.2	Dys,CD	2.8	HD,CHD	3.7
	8	Hyp,HD	3.0	Dys,CLD	2.8	Hyp,HD	3.2
	9	PVD,CD	2.9	Hyp,HD	2.7	PVD,CD	3.1
	10	Dys,CLD	2.8	HD,CHD	2.7	Hyp,PD	3.0
quartet	1	Hyp,HD,CHD	2.5	Hyp,Dys,CD	2.3	Hyp,HD,CHD	2.8
	2	Hyp,PVD,CD	2.4	Hyp,PVD,CD	2.2	Hyp,PVD,CD	2.5
	3	Hyp,Dys,CD	2.2	Hyp,HD,CHD	2.2	Hyp,Dys,CD	2.1
	4	Hyp,PVD,Dys	1.9	Hyp,Dys,CLD	1.9	Hyp,CKD,CD	1.9
	5	Hyp,Dys,CLD	1.8	Hyp,PVD,Dys	1.9	Hyp,CHD,CD	1.9
	6	Hyp,CHD,CD	1.8	Hyp,CHD,CD	1.8	Hyp,PVD,Dys	1.9
	7	Hyp,PVD,CHD	1.7	Hyp,Dys,CHD	1.7	Hyp,Dys,CLD	1.8
	8	Hyp,Dys,CHD	1.7	Hyp,PVD,CHD	1.6	Hyp,PVD,CHD	1.8
	9	Hyp,CKD,CD	1.6	Hyp,PVD,CLD	1.5	Hyp,CKD,CHD	1.8
	10	Hyp,PVD,CLD	1.6	Hyp,CLD,CD	1.5	Hyp,CKD,PVD	1.7

DM in the table is omitted. *Hyp* hypertension, *Dys* dyslipidemia, *CVD* cerebrovascular disease, *CLD* chronic liver disease, *CHD* coronary heart disease, *CKD* chronic kidney disease, *PVD* peripheral vascular disease, *HD* heart disease, *Arr* arrhythmia, *Cho* cholelithiasis, *SC* senile cataract, *Ane* anemia

**Table 3:** Prevalence of the 10 most common morbidities about DM by age

Type	Age	50–59		60–69		70–79		≥ 80	
	Order	NCDs	%	NCDs	%	NCDs	%	NCDs	%
dyads	1	Hyp	9.6	Hyp	14.9	Hyp	20.4	Hyp	18.8
	2	Dys	7.4	Dys	7.6	CVD	10.7	CVD	10.8
	3	CLD	6.4	CVD	7.1	CHD	9.2	CHD	9.8
	4	CKD	4.6	CLD	6.4	PVD	8.6	PVD	7.9
	5	CVD	4.2	PVD	6.1	Dys	7.4	CKD	7.8
	6	PVD	4.0	CHD	5.6	CKD	7.1	HD	7.2
	7	CHD	3.1	CKD	5.6	CLD	6.2	Arr	5.7
	8	gout	2.7	HD	3.6	HD	6.0	Dys	5.4
	9	HD	2.1	gout	3.3	Arr	4.5	CLD	4.6
	10	Cho	1.8	SC	2.5	gout	4.3	Ane	4.5
triads	1	Hyp,Dys	4.0	Hyp,CVD	5.5	Hyp,CVD	9.0	Hyp,CVD	9.4
	2	Dys,CLD	3.3	Hyp,Dys	5.2	Hyp,CHD	7.6	Hyp,CHD	8.4
	3	Hyp,CLD	3.3	Hyp,PVD	4.4	Hyp,PVD	7.0	Hyp,PVD	6.7
	4	Hyp,CVD	3.0	Hyp,CHD	4.3	Hyp,Dys	5.9	Hyp,CKD	6.7
	5	Hyp,CKD	2.8	Hyp,CLD	4.1	Hyp,CKD	5.8	Hyp,HD	6.2
	6	Hyp,PVD	2.4	Hyp,CKD	4.0	HD,CHD	5.2	HD,CHD	6.1
	7	PVD,Dys	2.1	HD,CHD	3.0	Hyp,HD	5.0	CHD,CVD	4.9
	8	Hyp,CHD	2.1	Dys,CVD	3.0	PVD,CVD	4.9	PVD,CVD	4.9
	9	Dys,CVD	2.0	Dys,CLD	2.9	Hyp,CLD	4.8	Hyp,Arr	4.8
	10	CKD,CLD	2.0	PVD,CVD	2.8	CHD,CVD	4.0	Hyp,Dys	4.6
quartet	1	Hyp,Dys,CLD	1.8	Hyp,HD,CHD	2.3	Hyp,HD,CHD	4.3	Hyp,HD,CHD	5.2
	2	Hyp,Dys,CVD	1.5	Hyp,Dys,CVD	2.3	Hyp,PVD,CVD	4.3	Hyp,PVD,CVD	4.4
	3	Hyp,PVD,Dys	1.3	Hyp,PVD,CVD	2.3	Hyp,CHD,CVD	3.5	Hyp,CHD,CVD	4.4
	4	Hyp,CKD,Dys	1.2	Hyp,PVD,Dys	2.0	Hyp,Dys,CVD	3.3	Hyp,CKD,CHD	3.5
	5	Hyp,HD,CHD	1.1	Hyp,Dys,CLD	1.9	Hyp,PVD,CHD	3.1	Hyp,CKD,CVD	3.5
	6	Hyp,CKD,CLD	1.1	Hyp,PVD,CLD	1.7	Hyp,PVD,Dys	2.7	Hyp,PVD,CHD	3.5
	7	PVD,Dys,CLD	1.1	Hyp,Dys,CHD	1.7	Hyp,CKD,CVD	2.6	Hyp,HD,CVD	3.3
	8	Hyp,PVD,CLD	1.1	Hyp,CHD,CVD	1.6	Hyp,CKD,CHD	2.6	Hyp,Arr,CHD	3.1
	9	Hyp,PVD,CVD	1.1	Hyp,CLD,CVD	1.5	Hyp,Dys,CHD	2.5	HD,CHD,CVD	3.1
	10	Hyp,Dys,gout	1.1	Hyp,CKD,Dys	1.5	Hyp,CKD,PVD	2.4	Hyp,CKD,HD	3.0

DM in the table is omitted. *Hyp* hypertension, *Dys* dyslipidemia, *CVD* cerebrovascular disease, *CLD* chronic liver disease, *CHD* coronary heart disease, *CKD* chronic kidney disease, *PVD* peripheral vascular disease, *HD* heart disease, *Arr* arrhythmia, *Cho* cholelithiasis, *SC* senile cataract, *Ane* anemia

The most common NCDs included hypertension, dyslipidemia, CVD, CLD, PVD, CHD, CKD, HD, gout, arrhythmia, anemia, and prostate disease (PD). The proportion of men with triad combinations including DM was generally higher than that of women with the same combinations (*P* < 0.001). Among quartets of NCD combinations, the 10 most common combinations differed by sex and age (*P* < 0.001).

### Multimorbidity patterns in people with DM

**Fig. 2:**
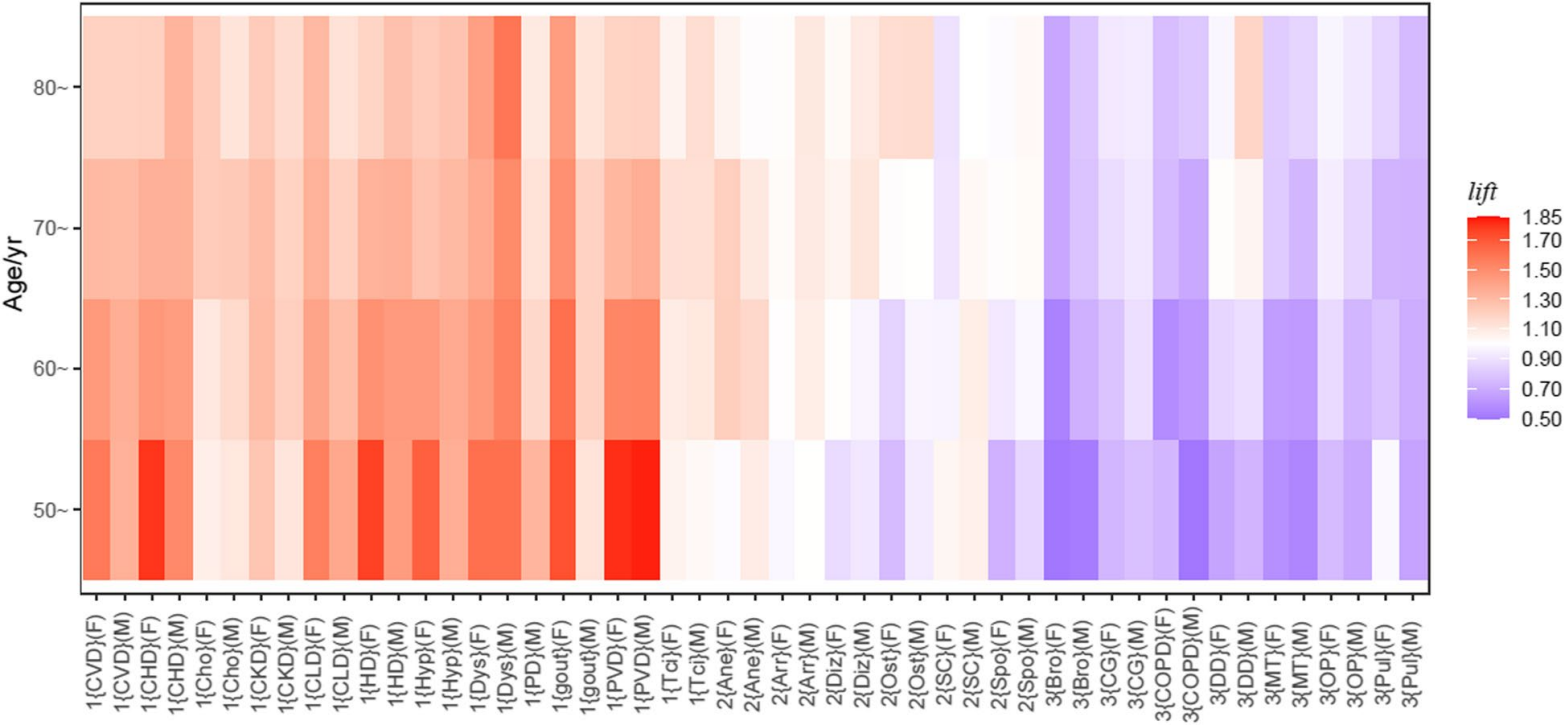
Heatmap of $lift$ values between 25 NCDs and DM for 8 age-sex–based subgroup ($sup>$.005). The red grid represents a $lift$ > 1, the redder the color, the greater the $lift$; the blue grid represents a $lift$ < 1, the bluer the color, the smaller the $lift$; and the white grid represents a $lift$ close to 1. The y-axis represents the age group. The 1 represents a positive association between NCD and DM. The 2 represents a weak or no association between NCD and DM. The 3 represents a negative association between NCD and DM. The F represents the female group. The M represents the male group. (CVD: cerebrovascular disease; CHD: coronary heart disease; Cho: cholelithiasis; CKD: chronic kidney disease; CLD: chronic liver disease; HD: heart disease; Hyp: hypertension; Dys: dyslipidemia; PD: prostate disease; PVD: peripheral vascular disease; Tci: transient cerebral ischemia; Ane: anemia; Diz: dizziness/vertigo; Ost: osteoarthropathy; SC: senile cataract; Spo: spondylosis: Bro: bronchiectasis; CG: chronic gastritis; COPD: chronic obstructive pulmonary disease; DD: disc degeneration; MT: malignant tumor; OP: osteoporosis; Pul: pulmonary heart disease)

**Fig. 3:**
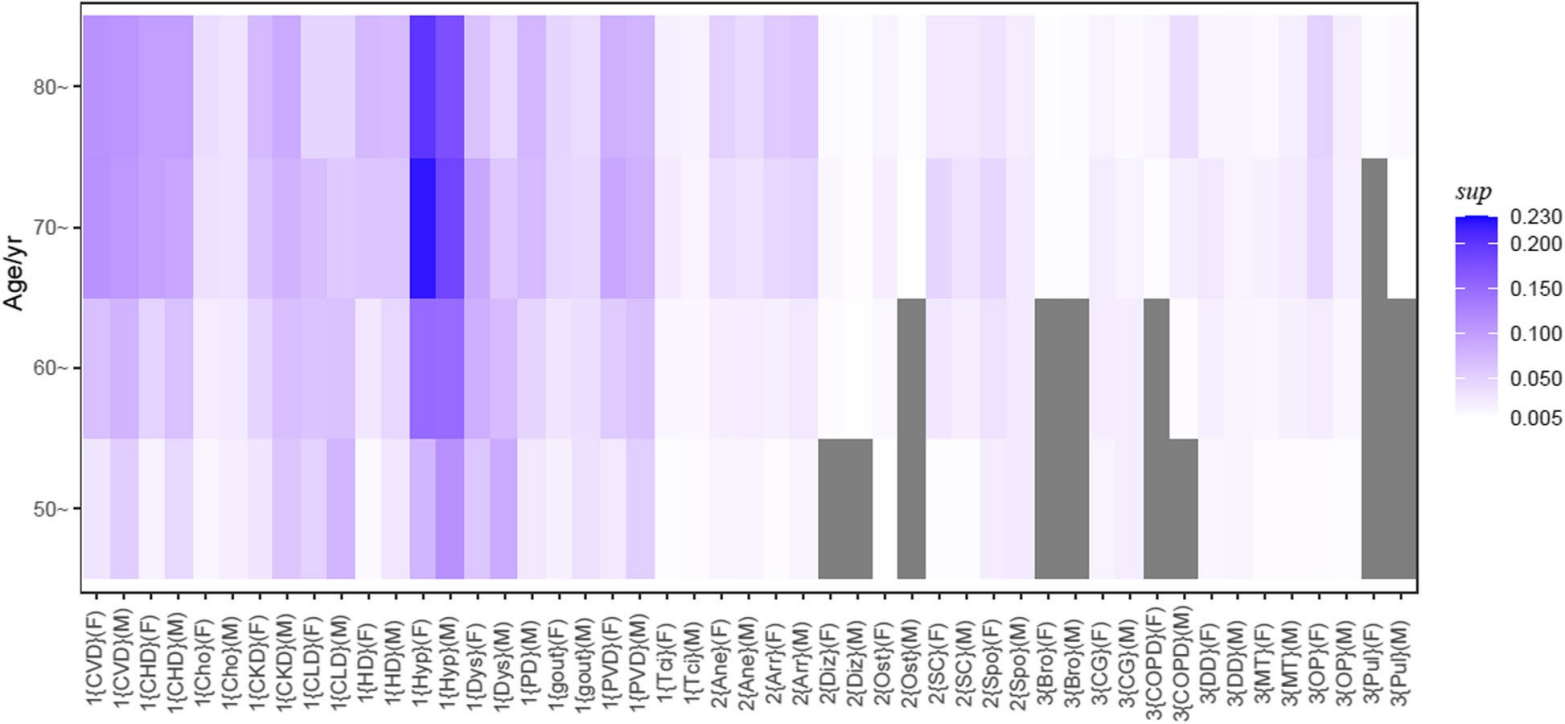
Heatmap of $sup$ values between 25 NCDs and DM for 8 age-sex–based subgroup ($sup>$.005). The blue grid represents the $sup$ value, the bluer the color, the greater $sup$; the gray grid represents a $sup$ < .005. The y-axis represents the age group. The 1 represents a positive association between NCD and DM. The 2 represents a weak or not association between NCD and DM. The 3 represents a negative association between NCD and DM. The F represents the female group. The M represents the male group. (CVD: cerebrovascular disease; CHD: coronary heart disease; Cho: cholelithiasis; CKD: chronic kidney disease; CLD: chronic liver disease; HD: heart disease; Hyp: hypertension; Dys: dyslipidemia; PD: prostate disease; PVD: peripheral vascular disease; Tci: transient cerebral ischemia; Ane: anemia; Diz: dizziness/vertigo; Ost: osteoarthropathy; SC: senile cataract; Spo: spondylosis: Bro: bronchiectasis; CG: chronic gastritis; COPD: chronic obstructive pulmonary disease; DD: disc degeneration; MT: malignant tumor; OP: osteoporosis; Pul: pulmonary heart disease)

In this part, the minimum $sup$ threshold was set as 0.005, to get as many association rules as possible. The reason for not setting $sup$=0.001 is to avoid a large error in $lift$. The number of items of the association rule was set to 2, and the "consequent" of the association rule was set to "DM". When $lift>1.1$, the ARM showed a list of NCDs that were positively related to DM, while $lift<0.9$ created a list of NCDs that were negatively related to DM. The remaining NCDs were weakly related or not related to DM. After analysis, there was a total of 25 NCDs. Figures 2 and 3 show the results of the ARM by $lift$ and $sup$, respectively.

There were 12 NCDs positively related to DM. The proportion of people with most of these NCDs in combination with DM increased with age. The proportions increased more in women than in men. The {PVD, DM} had the highest $lift$ in the group of 50 − 59 years. The {dyslipidemia, DM} and {gout, DM} had the highest $lift$ value in people 60 years or older in the male and female groups, respectively.

Anemia, arrhythmia, dizziness/vertigo, osteoarthropathy, senile cataract and spondylosis were in the weakly related or not related group.

A total of 7 NCDs were negatively related to DM. The co-occurrence of these NCDs in DM patients is unlikely to be due to randomness. In the subgroups of sex and age, disc degeneration appeared in both the positively related and negatively related groups.

In order to support the reliability of the conclusions, Chi-square tests were used to assess the statistical significance of the association rules. This includes 12 positively associated NCDs and 7 negatively associated NCDs. Odds ratios (OR) and 95% confidence intervals (95% CI) of associations between antecedent NCDs and DM in the association rules are shown in Table 4. For 12 NCDs in the positive group, all the ORs are greater than 1, and *P* < 0.001, indicating that the DM was more likely to be positive when the combinations of antecedent NCDs were positive than negative. For the 7 NCDs in the negative group, the results were reversed.

**Table 4:** OR and 95% CIs of the associations between NCDs and DM

	DM(*n*)	non-DM(*n*)	95%CI OR (lower, upper)		OR	*P* value
CVD	21,695	46,452	1.791	1.860	1.826	< .001
non-CVD	49,237	192,459				
CHD	17,762	34,748	1.923	2.003	1.963	< .001
non-CHD	53,170	204,163				
cholelithiasis	7469	20,588	1.214	1.283	1.248	< .001
non-cholelithiasis	63,463	218,323				
CKD	17,731	45,739	1.380	1.436	1.408	< .001
non-CKD	53,201	193,172				
CLD	19,069	42,758	1.687	1.654	1.720	< .001
non-CLD	51,863	196,153				
HD	11,773	23,591	1.773	1.860	1.816	< .001
non-HD	59,159	215,320				
hypertension	43,976	92,997	2.516	2.604	2.560	< .001
non-hypertension	26,956	145,914				
dyslipidemia	22,518	42,870	2.087	2.168	2.127	< .001
non-dyslipidemia	48,414	196,041				
PD	7260	18,215	1.343	1.422	1.382	< .001
non-PD	63,672	220,696				
gout	10,383	23,613	1.564	1.643	1.603	< .001
non-gout	60,549	215,298				
PVD	18,233	33,076	2.109	2.198	2.153	< .001
non-PVD	52,699	205,835				
TCI	4036	11,781	1.121	1.207	1.163	.001
non-TCI	66,896	227,130				
bronchiectasis	1269	7082	0.537	0.606	0.571	< .001
non-bronchiectasis	69,663	221,829				
CG	564	2155	0.802	0.966	0.881	< .001
non-CG	70,368	236,756				
COPD	2338	11,570	0.640	0.701	0.670	< .001
non-COPD	68,594	227,341				
DD	5210	22,655	0.733	0.781	0.757	< .001
non-DD	65,722	216,256				
MT	4427	24,609	0.561	0.599	0.580	< .001
non-MT	66,505	214,302				
osteoporosis	5821	23,300	0.803	0.853	0.827	< .001
non-osteoporosis	65,111	215,611				
PHD	922	4155	0.692	0.800	0.744	< .001
non-PHD	70,010	234,756				

*CVD* cerebrovascular disease, *CHD* coronary heart disease, *CKD* chronic kidney disease, *CLD* chronic liver disease, *HD* heart disease, *PD* prostate disease, *PVD* peripheral vascular disease, *TCI* transient cerebral ischemia, *CG* chronic gastritis, *COPD* chronic obstructive pulmonary disease, *DD* disc degeneration, *MT* malignant tumor, *PHD* pulmonary heart disease

### Variations in DM multimorbidity patterns by sex and age

**Fig. 4:**
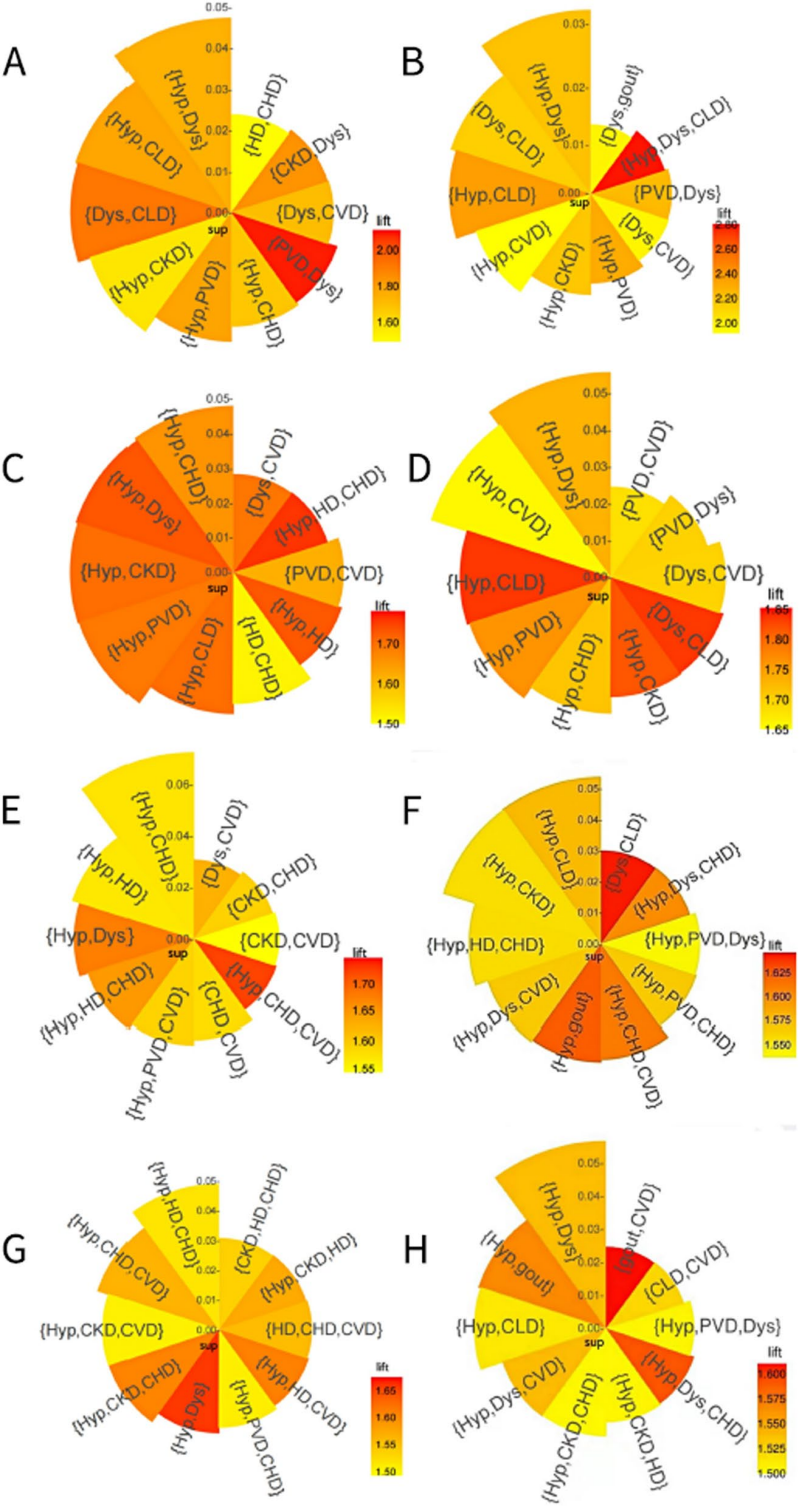
The 10 largest $lift$ rules about DM. Increased length of the sector indicates a larger $sup$. The darker the color, the higher the $lift$. **A**: male 50–59 years; **B**: female 50–59 years; **C**: male 60–69 years; **D**: female 60–69 years; **E**: male 70–79 years; **F**: female 70–79 years; **G**: male 80 ~ years; **H**: female 80 ~ years; *be clearer on age range of G and H*. (Hyp: hypertension; Dys: dyslipidemia; CVD: cerebrovascular disease; CLD: chronic liver disease; CHD: coronary heart disease; CKD: chronic kidney disease; PVD: peripheral vascular disease; HD: heart disease)

For the selection of combinations that were important and closely related, we set $sup\ge$0.01, $lift\ge$ 1.5, and the $con$ was unbounded. Among the four age groups of men, 48, 81, 137 and 108 rules were detected, while 16, 53, 136 and 115 rules were found in women. The top 10 association rules with larger $lift$ in 8 subgroups are described in Fig. 4. The association rules include a total of 9 NCDs. The types and order of the most common NCDs are quite different between age-sex subgroups. Multimorbidity in patients with DM was more prominent in men and older individuals. Most of men’s sup were higher than women’s, especially in the group of 50 − 59 years. CHD occurred more frequently in men than in women and more frequently in the group of 70 years and older than in group of 50 − 69 years. In addition, 4 rules included gout in women, but zero in men. Most of the rules in the groups younger than 70 years were triads, while most of the rules were quartets in the groups of 70 years and older. Hypertension, CLD and dyslipidemia appears more frequently in the association rules for the groups younger than 70 years. The DM multimorbidity network was complex in the group of 70 years and older. CKD, CVD, CHD and HD frequently appeared in the association rules.

## Discussion

This study examined the relationship between 74 NCDs and DM using ARM as a novel classification method. Of the 74 NCDs, 12 were positively associated with DM, and 7 were negatively associated. We also used ARM to explore the DM multimorbidity pattern in age-sex subgroups, with 9 common NCDs included in the results. Men and older people were more vulnerable to multimorbidity in those with DM, and particular multimorbidities in people with DM cluster together frequently and more often than expected by chance. CVD, CHD, CKD, CLD, HD, hypertension, dyslipidemia, gout, and PVD were common in the DM multimorbidity network and were directly or indirectly related to DM. Hypertension, CLD and dyslipidemia were more common in people younger than 70 years, while CKD, CVD, CHD and HD were more common in people older than 70 years.

Among the positive correlation group, the shared etiologies of most NCDs and DM have been demonstrated in previous studies, such as for CVD [37], CHD [38, 39], CKD [40], CLD [41], heart disease [42], dyslipidemia [43, 44], prostate disease [45], gout [46], PVD [47], and transient cerebral ischemia [48]. Further explanations of the clinical significance are as follows. A study of 2,400 older people with and without DM confirms that DM is significantly associated with brain infarction [37]. A genome-wide, multi-ancestry study of genetic variation for DM and CHD shows that a genetically mediated increase in DM risk confers a higher risk of CHD [40]. A 10-year follow-up study showed that DM is associated with higher risks of liver cancer and CLD [41]. Epidemiologic and clinical data from the last 2 decades have shown that the prevalence of heart failure in DM is very high and that the prognosis for patients with heart failure is worse in those with DM than in those without it [42]. Data from animal models and humans show that very low levels of high-density lipoprotein cholesterol are often associated with hyperglycaemia and DM. Cholesterol homeostasis is important for adequate beta-cell insulin secretion [45]. Data from Francesco et al. suggest that metabolic alterations and CVD influence aggressive and metastatic prostate cancer [45]. A genome-wide analysis study showed that after excluding obesity and alcohol consumption behaviour, this study showed that patients with gout and DM share the common genetic factors most, and that there is a mutual inter-dependent effect on higher incidences [46].

The {dyslipidemia, DM} and {gout, DM} had the greatest $lift$ in men and women, respectively. The relationship between dyslipidemia, gout and DM in men and women needs to be considered. The {cholelithiasis, DM} occurred at least 1.2 times more than expected under statistical independence. This was likely as cholelithiasis and DM have the same pathological pathways or potential risk factors. However, previous multimorbidity studies have not found this relationship. There needs to be more clarity in understanding the co-occurrence of cholelithiasis and DM. A study showed that cholelithiasis was directly related to body weight and abdominal adiposity[49]. Obesity is related to DM, suggesting a potential relationship between cholelithiasis and DM. Clarifying the relationship between cholelithiasis and DM is of great significance for patients.

Seven NCDs were negatively related to DM. There have been limited studies on chronic gastritis, malignant tumor, osteoporosis, bronchiectasis and pulmonary heart disease with DM. A review study [50] listed seven studies on DM and disc degeneration, of which four showed that DM was a significant risk factor for disc degeneration, and the remaining three failed to find any association. Another study concluded that DM has a devastating effect on disc degeneration [51]. Our results showed a negative association between disc degeneration and DM, adding to the clinical evidence that is not consistent. The published studies on the co-occurrence of COPD and DM are controversial [52, 53]. There may be some potential influencing factors of COPD and DM, resulting in a negative association between COPD and DM. The biological link between COPD and DM is still unclear.

Our other goal was to explore the association rules between 74 NCDs and DM by subgroup analyses. Compared with the existing literature, this study focused on the DM multimorbidity network rather than that of all included NCDs. Our results are more detailed and comprehensive. In the published studies, only several rules on DM were generated, and most of them were already well-known, such as {hypertension, DM} [7, 15, 17–19, 21, 54–56], {dyslipidemia, DM} [7, 15, 17–19, 56], {CHD, DM} [12, 19, 57], {CKD, DM}[12]. Our results highlight some important combinations with DM and show differences in the type and order of the most common associations by sex and age, which is consistent with the results of Han et al.[55]. Multimorbidity in patients with DM are more prominent in men and older people. There were significant differences in {gout, DM} and {dyslipidemia, DM} for men and women. Gout was more strongly related to DM in women than that in men, meaning there is a higher risk for women. Among men, the most common rule for dyslipidemia appeared in {hypertension, dyslipidemia, DM} with large $sup$. Among women, dyslipidemia was more likely to be related to other NCDs than that in men. The proportion of people with multimorbidity in those with DM increased with advancing age, but it was lower in those older than 80 years compared to those aged 70 to 79 years. For those younger than 70 years, triad was the most common type of rule. Hypertension, dyslipidemia and CLD are common NCDs. They play an important part in multimorbidity in patients with DM, while CKD, CVD, CHD and heart disease frequently co-occur in people with DM older than 70 years. It suggests that screening for additional NCDs in each age group in a targeted manner becomes more efficient. The difference in age and sex may be explained by survival bias.

Several limitations of our study need to be acknowledged. First, our research data were from hospitalized patients, therefore the proportions of people with multimorbidities cannot be applied to the whole population. However, this was separate from the main aim of this study which was to focus on the DM multimorbidity network. In addition, our results based on more severe cases may provide ideas for research into the early prevention of combinations of DM and other NCDs. Second, due to the cross-sectional nature of the data the results did not demonstrate causal links between NCDs and DM. Finally, the time of DM onset and detailed information on patients’ physical strategies, lifestyle factors, socioeconomic status and family history were not included in the model in this analysis due to lack of data availability, and the data set anonymized participants to avoid possible misuse; therefore, some potential confounding factors were not taken into consideration.

Despite the increasing prevalence of multimorbidity in patients with DM, there are no specific recommendations for diagnosis and treatment [58]. The management and prevention of DM with multimorbidity through health interventions should be offered to individuals by the primary health care providers. However, there is a lack of evidence of effective interventions in previous studies. Investigating the DM multimorbidity network remains an area that needs to be explored in future research [59]. Our results confirm and expand the findings of previous studies on multimorbidity in patients with DM. Because of the large sample size in this study, our results are generally more reliable than those in previous studies. These results have the potential to consider the DM multimorbidity network as a framework for addressing the care of older adults with DM multimorbidity, and to support policies for the management of DM patients with multimorbidity in primary care and community settings. The results also provide support and a new perspective for future longitudinal or experimental studies to identify potential mechanisms and risk factors for the DM multimorbidity network. This will help healthcare providers improve the effectiveness of DM management. In addition, different strategies should be developed to prevent multimorbidity in people with DM. When developing guidelines for the management of DM patients, age, sex and potential risks of diseases need to be taken into account for recommendations on the diagnosis and monitoring.

## Conclusion

Our results indicate that the DM multimorbidity network varies by age and sex. It suggests that targeted screening for DM according to age and sex will increase efficiency. The combination {cholelithiasis, DM} gave an unexpected multimorbidity score and represented a complex comorbid condition. Of course, further longitudinal or experimental studies are needed to establish causal relationships between NCDs and DM. A more integrated multidisciplinary approach focusing on improved management and prevention of DM may help prevent other NCDs in the network. The guidelines on the management of patients with DM should be focused on recommendations based on age and sex and potentially revised to consider the co-management of NCDs that cluster around DM.

### Supplementary Information

Supplementary Material 1.

## Data Availability

The data sets generated during and analyzed in this study are not publicly available, as the hospital data cannot allowed to be disclosed, but are available from the corresponding author on reasonable request.

## Abbreviations

ARM: Association rule mining
NCD: Chronic non-communicable diseases
ICD-10: International classification of diseases version 10
DM: Diabetes mellitus
CVD: Cerebrovascular disease
CHD: Coronary heart disease
Cho: Cholelithiasis
CKD: Chronic kidney disease
CLD: Chronic liver disease
HD: Heart disease
Hyp: Hypertension
Dys: Dyslipidemia
PD: Prostate disease
PVD: Peripheral vascular disease
TCI: Transient cerebral ischemia
Ane: Anemia
Diz: Dizziness/vertigo
Ost: Osteoarthropathy
SC: Senile cataract
Spo: Spondylosis
Bro: Bronchiectasis
CG: Chronic gastritis
COPD: Chronic obstructive pulmonary disease
DD: Disc degeneration
MT: Malignant tumor
OP: Osteoporosis
Pul: Pulmonary heart disease
