# Supplementary material for: The diabetes mellitus multimorbidity network in hospitalized patients over 50 years of age in China: data mining of medical records
Source: BMC Public Health. 2024 May 29;24:1433. doi: 10.1186/s12889-024-18887-y (PMC11134652; doi:10.1186/s12889-024-18887-y)
Supplement: Supplementary file 1 — Supplementary Material 1. [file 12889_2024_18887_MOESM1_ESM.docx]

**ICD-10 numbers of 75 NCDs included in the analysis**

| ICD-10 | NCDs |
| --- | --- |
| I10-I15 | Hypertension |
| R52.1-R52.2 | Chronic pain |
| E10-E14 | Diabetes mellitus |
| E78 | Lipoprotein metabolism disorder |
| K30 | Indigestion |
| K29.3-K29.5 | Chronic gastritis |
| I20-I25 | Coronary heart disease |
| J44 | Chronic obstructive pulmonary disease |
| I60-I69 | Cerebrovascular disease |
| N03,N04,N06,N07,N08,N11,N13,N14-16，N18、N19、N20-23、N25-29 | Chronic kidney disease |
| K80 | Gallstone disease |
| K81.1 | Chronic cholecystitis |
| D73 | Spleen disease |
| I70-I73.9 | Peripheral vascular disease |
| I83 | Varicose veins |
| F20 | Schizophrenia |
| F31 | Bipolar disorder |
| C00-C97 | Malignant tumor |
| F00-F03 | Dementia |
| G30 | Alzheimer’s disease |
| J47 | Bronchiectasis |
| H40-H42 | Glaucoma |
| H25,H28.0-H28.2 | Senile cataract |
| J45-J46 | Asthma |
| J32 | Chronic sinusitis |
| J31.0 | Chronic rhinitis |
| J31.1 | Chronic nasopharyngitis |
| J31.2 | Chronic pharyngitis |
| B18 | Chronic viral hepatitis |
| J37.0 | Chronic laryngitis |
| J37.1 | Chronic laryngotracheitis |
| K57 | Diverticulosis of intestine |
| E02-E03 | Hypothyroidism |
| E05 | Hyperthyroidism |
| H90-H91 | Hearing loss |
| H93.1 | Tinnitus |
| H53-H54 | Vision impairment or blindness |
| L40 | Psoriasis |
| L20-L30 | Dermatitis and eczema |
| D50-D64 | Anemia |
| N40,N41.1-3,N41.8-9,N42 | prostate disease |
| G43,G44 | Migraine |
| K70,K71.3-K71.5,K71.7,K72.1,K73-76 | Chronic liver disease |
| F32-F33 | Depression |
| G40 | Epilepsy |
| F40,F41 | Anxiety |
| G20-G22 | Parkinson’s disease |
| K59.0 | Constipation |
| J35.0 | Chronic tonsillitis |
| F50 | Eating disorders |
| F51,G47 | Sleep disorder |
| I05-I09 | Chronic rheumatic heart disease |
| I50 | [Heart failure](javascript:;) |
| I51.9 | Heart disease (not specifically) |
| I44-I49 | Arrhythmia |
| K25.4-K25.9 | Chronic gastric ulcer |
| K26.4-K26.9 | Chronic duodenum ulcer |
| K27.4-k27.9 | Chronic peptic ulcer |
| K28.4-K28.9 | Chronic gastrojejunal ulcer |
| M05-M06,M79.0 | Rheumatoid arthritis |
| E79,M10 | Gout |
| M80-M82 | Osteoarthropathy |
| E66 | Obesity |
| G45 | Transient cerebral ischemia |
| M15-M19 | Srthropathy |
| I26-I27 | Pulmonary heart disease |
| M30-M36 | Systemic connective tissue disease |
| M45-M49 | Spondylosis |
| M50-M51 | Intervertebral disc degeneration |
| I34-I37 | [Valvulopathy](javascript:;) |
| K00.0 | Edentulous |
| F45 | Somatoform disorder |
| G80-G83 | Paralysis |
| R42,H81.0-H82 | Dizziness/vertigo |
| N39.3-N39.4,R32 | Urinary incontinence |
| G50-G64 | Neurological disorders |
